# Transanal advancement flap in a female patient with anterior transsphincteric anal fistula: a video vignette

**DOI:** 10.1007/s10151-025-03175-7

**Published:** 2025-09-20

**Authors:** Y. Yildirim, A. S. Akgun, C. Arslan

**Affiliations:** 1 Department of General Surgery, Istanbul Medipol University, 34320 Istanbul, Turkey; 2 Department of Radiology, Istanbul Medipol University, 34320 Istanbul, Turkey

An anal fistula is an inflammatory pathway between the anal canal and the perianal skin. Infection from the anal glands, which form a cryptoglandular abscess at the dentate line and then communicate with the perianal skin, is thought to be the underlying cause. The incidence of anal fistula in European countries is 1.2–2.80 per 10,000 people, and severe pain, bleeding, and purulent discharge are the main symptoms [1]. Anal fistulas are categorized as “simple” or “complex” according to the classification of the American Society of Colon and Rectal Surgeons (ASCRS), which is more effective for surgical clinical use [2]. Complex anal fistulas include high transsphincteric, suprasphincteric, extrasphincteric fistulas that involve > 30% of the external anal sphincter (EAS) or those associated with inflammatory bowel disease, radiation, malignancy, or horseshoe fistulas, anterior fistulas in women, and fistulas with secondary tracts. While simple fistula can be managed with fistulectomy without any complication, complex fistulas can be challenging for surgeons. The aim of complex fistula treatment is to minimize recurrence and to prevent incontinence. Many techniques have been described for this purpose; however, there is no widely accepted one.

The transanal advancement flap is a feasible and safe sphincter-preserving procedure with reported rates of up to approximately 80% healing overall and 64% healing even in Crohn’s fistulas with acceptable incontinence rates [3]. These recovery rates are supported by meta-analysis and reviews [4, 5].

We present the case of a 43-year-old female patient with perianal pain and fever. Clinical examination revealed an external fistula opening located on the left anterior side, accompanied by fluctuation in the perineal region. MRI revealed a transsphincteric anal fistula with an internal opening at the anterior midline at the level of the dentate line, accompanied by an abscess extending to the vagina on the left anterior side. The abscess was drained, and a seton was placed. Two months later, once the infection had resolved, the patient underwent a full-thickness transanal flap; this is demonstrated step by step in the following video (link below).

## Supplementary Information

Below is the link to the electronic supplementary material.

Supplementary file1 (MP4 878880 KB)

## Data Availability

No datasets were generated or analysed during the current study.
